# Gender Bias and Conversational Agents: an ethical perspective on Social Robotics

**DOI:** 10.1007/s11948-022-00376-3

**Published:** 2022-04-21

**Authors:** Fabio Fossa, Irene Sucameli

**Affiliations:** 1 Department of Mechanical Engineering, Politecnico di Milano, Milano, Italy; 2 Department of Computer Science, University of Pisa, Pisa, Italy

**Keywords:** Design Ethics, Conversational agents, Gender Bias, Discrimination, Moral Technology

## Abstract

The increase in the spread of conversational agents urgently requires to tackle the ethical issues linked to their design. In fact, developers frequently include in their products cues that trigger social biases in order to maximize the performance and the quality of human-machine interactions. The present paper discusses whether and to what extent it is ethically sound to intentionally trigger gender biases through the design of virtually embodied conversational agents. After outlining the complex dynamics involving social biases, social robots, and design, we evaluate the ethics of integrating gender cues in conversational agents, analysing four different approaches to the problem. Finally, we suggest which approach in our opinion might have the best chances to reduce the negative effects of biases and discriminatory visions of gender dynamics.

## Introduction

As artificial agents^1^ spread far and wide, it is important to critically assess related design choices from an ethical point of view. This is even more necessary with regard to the field of social robotics, where products are usually expected to interact with people in the smoothest, most engaging possible way. In particular, it is pivotal to ask to what extent it is ethically permissible to deliberately exploit pre-existing social biases in order to build products that successfully meet user expectations, blend in perfectly with their context of use, and maximize the perceived quality of interactions.

This paper presents a discussion on whether it is ethically sound to leverage pre-existing biases in order to maximize the quality of human-machine interactions. Since such an issue potentially pertains to the entire field of social robotics and might apply to all kinds of bias, narrowing the scope of the analysis is both necessary and likely to be more productive in terms of theoretical insights and practical applications. On this account, we clarify our more general claims by focusing on a specific type of application, i.e., virtually Embodied Conversational Agents (ECAs)^2^, and on a specific kind of bias, i.e., gender bias. Nevertheless, we believe that our argument may apply throughout the entire field of social robotics and hold for other kinds of biases as well.

As a result, the main question we consider is:

### Q

Is it ethically permissible to align the design of ECAs to gender biases in order to improve interactions and maximize user satisfaction?

To discuss Q, we carry out two main tasks. First, we review the literature dedicated to bias-related ethical issues with reference to ECAs in order to contextualize our research question and systematically account for the problems it addresses [Sect. 2]. Secondly, we introduce and ethically assess four possible answers, namely:

- A1: It is ethically *permissible* to exploit widespread gender biases in order to maximize the quality of interactions between ECAs and users [Sect. 3.1].
- A2: it is ethically *inadmissible* to exploit gender cues in ECA design, even when they help improve human-machine interaction [Sect. 3.2].
- A3: it is ethically permissible to insert gender cues into ECA design *as long as* those cues do not spread a discriminatory vision of gender dynamics [Sect. 3.3].
- A4: the inclusion of gender cues in ECA design should be used *proactively* to limit the spread of discriminatory bias and promote ethical attitudes [Sect. 3.4].

As a final step, we weigh the various positions against each other and submit our considered judgment on the issue [Sect. 4]. Such an inquiry represents an original contribution to the discussion which we hope will support the effort of aligning ECA technology to relevant ethical standards.

## Bias, Social Robots, and Ethics

This section introduces the main question we wish to explore by discussing the literature on bias in social robotics and its implications on design ethics. First (2.1), we clarify the role biases play with reference to social robots – and, more specifically, to ECAs (2.2) – by focusing the attention on the design strategy of *bias alignment*. Secondly, we underline its ethical significance by introducing the *feedback hypothesis*, both in general (2.3) and as it applies to ECAs (2.4). In light of this, we state our research question and set the stage for critically discussing four possible answers to it.

### Bias alignment

**Fig. 1:**
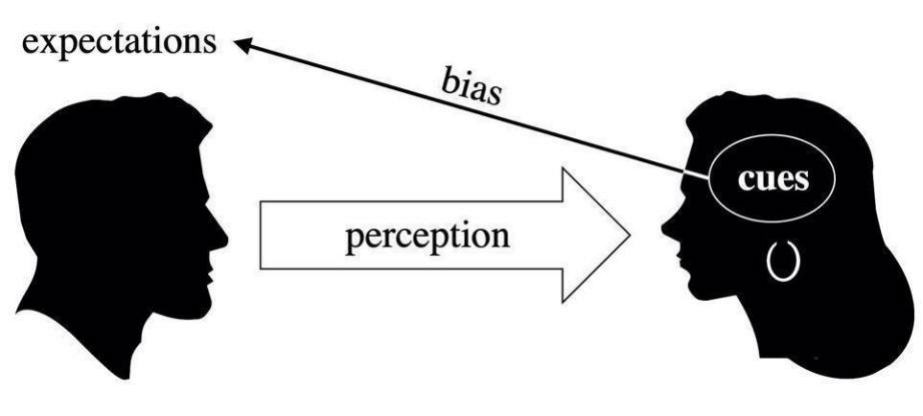
Bias Dynamics in Human-Human Interaction

Biases play a massive role in structuring human relations and the social life. Implicit assumptions based on approximate generalizations, rules of thumb and long-lasting habits influence the interpretation of our experience. In a sense, biases function as social scripts (De Angeli & Brahnam 2006) that assist us in coping with information incompleteness and complexity in everyday situations. As predetermined and rather fix schemes of information management, social biases – that is, biases that co-structure relations between humans – concern different aspects of the social sphere. However, the function they execute is similar. Basically, biases suggest associations between easily perceivable data or cues (such as ethnicity, age, gender, physical appearance, apparent economic status, social roles, specific tasks, and so on) and other, less immediately accessible but highly valuable pieces of information (such as competence, trustworthiness, intelligence, kindness, and so on). In doing so, biases concur to forming expectations and subsequent social behaviour (Fig. 1).

**Fig. 2:**
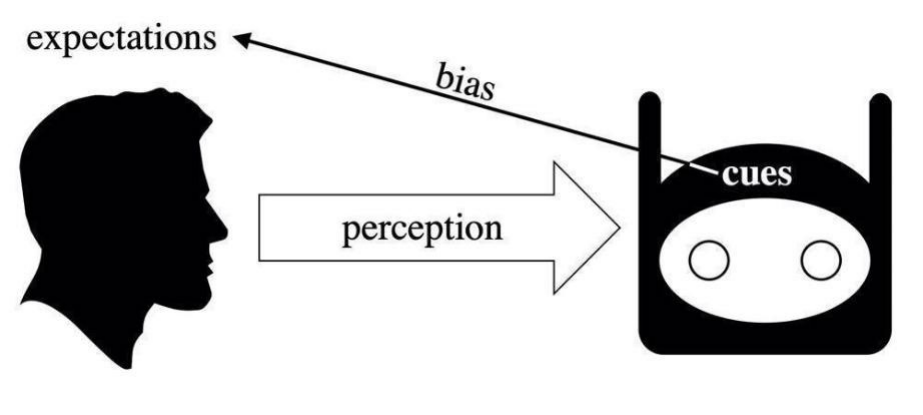
Bias Dynamics in Human-Machine Interaction

The fact that social roles and specific tasks are cues that trigger biased expectations is of particular importance. Indeed, it is reasonable to suppose that similar biased expectations will be triggered when artificial agents substitute human agents in given practical contexts. The automation of social roles and tasks might cause the same biased expectations influencing human social relations to be extended on to technological entities that are put in the place of humans. In many cases, this leads users to partially anthropomorphize artificial agents, projecting onto them typically human features such as gender, ethnicity, or social status. These projections, in turn, trigger biased information associations concerning competence, authority, trustworthiness, and other socially relevant features (Tay et al., 2014) (Fig. 2).

This intuition, that was firstly introduced and empirically confirmed in the human-computer interaction *Computers Are Social Actors* [CASA] studies (Nass et al., 1994; Nass et al., 1996), in recent years has been found to generally apply to human-robot interactions as well and has grown to become a widely accepted notion in social robotics (McDonnell & Baxter, 2019; Weßel et al., 2021). When social roles or tasks are automated, the way in which users respond to computer programs appear to be very similar to how they respond to social robots^3^.

The fact that social biases are relevant even in human-machine interactions is dense of design implications for social robotics. First of all, social biases have appeared to many in the social robotics community as conditions for designing successful interactions – which is arguably the central design goal in this field (Carpenter et al., 2009; Nomura, 2017). In light of their tremendous impact on user mental models, it seems reasonable to consider social biases as factors that massively influence the extent to which interactions are perceived as easy, pleasant, engaging, and effective. Ignoring their power would probably lead to interaction failures, suboptimal interaction quality and eventually rejections of the technology (Jung et al., 2016; Bryant et al., 2016).

The power of biases, however, is not just there to be coped with. It can be harnessed as well. Intelligently stimulating biases through the insertion of the right design cues might turn out to be the most successful strategy for optimizing interactions (Siegel et al., 2009). In other words, the cues that in human-human relations trigger biases that are conducive to effective interactions can be reproduced in the design of the artificial agent to which we want the corresponding human task to be delegated. Perceivable traits commonly associated with ethnicity, gender, age, physical beauty, and so on become tools in the designers’ hands. Through the adoption of various design cues, designers can influence the formation of user mental models of the technology and steer them towards desired outcomes, such as maximizing the feeling of trustworthiness suggested by a healthcare robot or the impression of competence produced by a smart assistant.

The practice of leveraging the power of biases in order to maximize the quality of interactions seems intimately connected to the idea that a seamless, almost “natural” introduction of artificial agents in the fabric of society must be promoted (Breazeal, 2003; Isaac & Bridewell, 2017; Eyssel & Kuchenbrandt, 2012). This implies a design strategy we call *bias alignment*. According to this strategy, artificial agents should be specifically designed to trigger the same social biases that are triggered in the corresponding human-human interaction that is being automated. In so doing, the unavoidable strangeness and difference of artificial agents will be countered, or covered, by their apparent social similarity to good old humans, so they will “fit in” just fine in the social context where they are deployed, without requiring extra cognitive efforts on the user part.

### Bias Alignment and ECAs

To further clarify these points, let us see how bias alignment works as applied to a specific class of social robots, i.e., Embodied Conversational Agents (ECAs).

ECAs are systems designed to mimic human-human interaction using natural language via text or voice. Moreover, ECAs are endowed with an embodied avatar representing an anthropomorphic body or part of it (Silvervarg et al., 2012). Both natural language processing and embodied avatars serve as platforms to incorporate different cues aimed at easing the interaction with human users. As explained, these elements facilitate the triggering of social biases that decrease the discomfort users experience when interacting with inanimate beings (Powers et al., 2005; Robertson, 2010; Jung et al., 2016; Kraus et al., 2018).

Gender biases offer themselves as a useful example of bias alignment with reference to ECAs. Gender attribution can be encouraged by-design through different cues that, as effectively described by Robertson (2010), serve as “cultural genitals”. In the absence of physical genitals, gender cues are proxies that trigger the identification of an ECA as a ‘malebot’ or a ‘fembot’^4^.

Gender attribution is rather easy to trigger, so multifarious cues based on traits that men and women are thought to have (descriptive stereotypes) or ‘should’ have (prescriptive stereotypes; cf. Brahnam & De Angeli, 2012) can be used. Some cues are physiognomic in nature: the hairstyle, the size of the eyes and of the head, the shoulder width, and the colour of the lips (De Angeli & Brahnam, 2006; Robertson, 2010; Eyssel & Hegel, 2012; Bernotat et al., 2017, 2021; Trovato et al., 2018). Even simple fashion accessories could suffice. In a work conducted by Jung et al., (2016), a male hat rather than a pair of pink earmuffs was enough for users to classify the ECA as respectively “male” and “female”.

Beyond the bodily domain, gender attribution can be triggered also through the bot’s tone of voice: e.g., fembots are usually endowed with high-pitch voices, which are commonly attributed to women (Robertson, 2010). Cues can be based on even more subtle proxies, such as specific skills or personal traits. For instance, the ‘masculinity’ or ‘femininity’ associated to a given task – e.g., changing a flat tyre vs. babysitting – will encourage users to project a specific gender to the system to which the task is delegated even if its avatar and voice are built to resist gender attribution. Indeed, gender identification is complex and involves multiple levels (Ladwig & Ferstl, 2018; Sutton, 2020).

Aligning the design of ECAs to users’ expectations through gender cues could be essential for acceptability. Gender biases triggered by design cues importantly influence user mental models and expectations. Different studies (Nass et al., 1997; De Angeli & Brahnam, 2006; Kraus et al., 2018) show that systems with male cues are seen as more dominant and assertive, while fembots are thought to be kinder, more communicative and helpful (Nass et al., 1994; Kuchenbrandt et al., 2014; Reich-Stiebert & Eyssel, 2017). Depending on the contexts of application, the power of gender biases could lead to adoption or disuse (Nass & Moon, 2000).

Consider this hypothetical scenario. Imagine that a company decides to develop a smart assistant to help people carrying out basic car maintenance tasks like changing the oil, a flat tire, or a consumed wiper. Let us also suppose that data collected in interviews show that the target customer group heavily associates the job as a mechanical with the male gender. Suppose further that previous psychological research showed that the male gender is commonly associated with higher degrees of authority, competence, and trustworthiness than the female gender. In this scenario, the bias alignment design strategy would suggest inserting some design cues that nudge users into projecting the male gender onto the technology.^5^ First, this will meet the users’ biased expectations concerning the ‘masculinity’ of the mechanical domain, ensuring a seamless introduction of the technology in its social context. Furthermore, this will leverage a double bias concerning competence and trustworthiness: (a) the bias according to which men are more competent than women when it comes to car maintenance; and (b) the bias according to which men are generally more authoritative and trustworthy than women. Adding a simple design cue to the technology like a low-pitched voice – which is commonly associated with the male gender – will do the trick.

By aligning design to social biases, their power will have been leashed and channelled towards the design goal of effective, pleasant, and smooth interaction. Stated in this teleological sense – i.e., according to a means-end approach where efficiency is at stake –bias alignment is a widely accepted design strategy in the social robotics community (Kraus et al., 2018; McDonnel & Baxter, 2019; Tay et al., 2014). As a recent UNESCO report (West et al., 2019) shows, for instance, feminine attributes are often used for the characterization of Personal Assistant (such as Alexa or Siri) so to nudge users into perceiving the system as more sympathetic and, at the same time, easier to control and dominate. This does not mean, however, that there is full agreement on the actual effectiveness of bias alignment (Sandry, 2015; Brahnam & Weaver, 2015; Reich-Stieber & Eyssel, 2017). Nevertheless, the dissident voices are considerably fewer than the agreeing ones.

### The Feedback Hypothesis

Through bias alignment, social biases can be leveraged to design better human-machine interactions. The benefits in terms of user acceptability are widely acknowledged. Are there any risks that should be considered?

As seen, implementing cues by design allows for social biases to be projected onto robots, so that users perceive them as familiar interlocutors. Interestingly, this implies that interactions with robots are intended to belong to the same practical category of interactions with humans. Accordingly, bias projection must be conceived as bidirectional as well. If biases triggered by humans transfer to corresponding interactions with ECAs, it is reasonable to assume that biases triggered by ECAs will *feedback* onto corresponding human relations as well. Let us call this claim the *feedback hypothesis*.

Even though, at least to our knowledge, there is no hard evidence supporting it, the feedback hypothesis has already been posited by some authors (Carpenter, 2009; Robertson, 2010; Sparrow, 2017; Bisconti, 2021) and appears to be conceptually sound enough for its potential impacts to be taken seriously. Impacts are relevant because, if the hypothesis holds, the social biases harnessed through design would be reinforced during interactions with robots and transferred back to humans. This would generate a lock-in situation where such biases, already deeply rooted in cultural views as they are, would become even further institutionalized and normalized, significantly increasing the amount of effort needed to eradicate them.

At first sight, it seems reasonable to claim that not every instance of bias leveraging is necessarily problematic from this perspective. For example, suppose that psychological research suggests that users tend to behave more calmly and collaboratively when they interact with artificial agents coloured in blue instead of red. As a consequence, a smart assistant is given a blue suit, and not a red one, to wear on its digital avatar body. No risk seems to arise.

Things are different when the leveraged biases have ethical significance, which is rather common when social relationships are concerned. For the purposes of our paper, suffice it to say that biases are unethical if they convey epistemically problematic beliefs which promote unacceptable discrimination, thus harming people by reducing their individuality to inconsistent and often spiteful generalizations - which is a clear offence to human dignity^6^.

As already said, many social biases are extensively based on highly sensitive perceptual data like ethnicity, skin colour, age, and gender. It is thus sensible to fear that leveraging discriminatory biases might enforce and solidify the connected forms of social discrimination, making it even harder to eradicate them and promote equal and fair social treatment. If this were the case, technology would become a booster of socially discriminatory biases and would engender a lock-in effect where pre-existing social biases would be stabilized and eventually normalized.

A lock-in effect involving discriminatory biases would be highly detrimental. Indeed, it would worsen the condition of both victims and perpetrators. Discriminated groups would have to face a silent, invisible but systemic diffusion of harmful biases. Users would be put in a position where the discriminatory biases they unintentionally share are opaquely strengthened and amplified through interactions with social technologies, working against their own moral aspirations.

In light of this, the best solution could be to separate risky biases from safe ones. Yet, it seems unlikely that biases can be absolutely categorized as discriminatory and non-discriminatory. Potentially discriminatory biases might be triggered through cues without engendering evident discriminatory outcomes. Gender biases are a good example here. Suppose, for instance, that conclusive evidence was found that gendered ECAs work better than non-gendered ones. Suppose further that a company is tasked to develop an ECA to offer customer service inside a women’s sauna. After some debate, the design team opts for a fembot, since they believe that, given the context of use, customers may react negatively to a malebot. In this case, we surmise, no unethical discrimination seems involved. Therefore, the way in which cues are implemented and the contexts of use affect the risks posed by design choices that exploit biases. It follows that the risks involved in bias alignment requires careful consideration so that the legitimate purpose of maximising interaction quality is pursued within the boundaries of what is ethically permissible.

Let us introduce another fictional example to better grasp the risks that the hypothesis wishes to stress. Suppose now that the team is appointed to design an ECA to carry out secretarial tasks assisting executives in a company where 80% of senior positions are held by white men aged between 55 and 65 years. Suppose further that psychological surveys showed that male subjects in the same age span tend to associate the secretary role with the female gender, while the male gender is associated with managerial roles. The team thus infers that a malebot would be perceived as strange if deployed to carry out secretary tasks, while a fembot would fit in just fine. Consequently, they implement gender cues studied to trigger the desired biases in the specific user group.

**Fig. 3:**
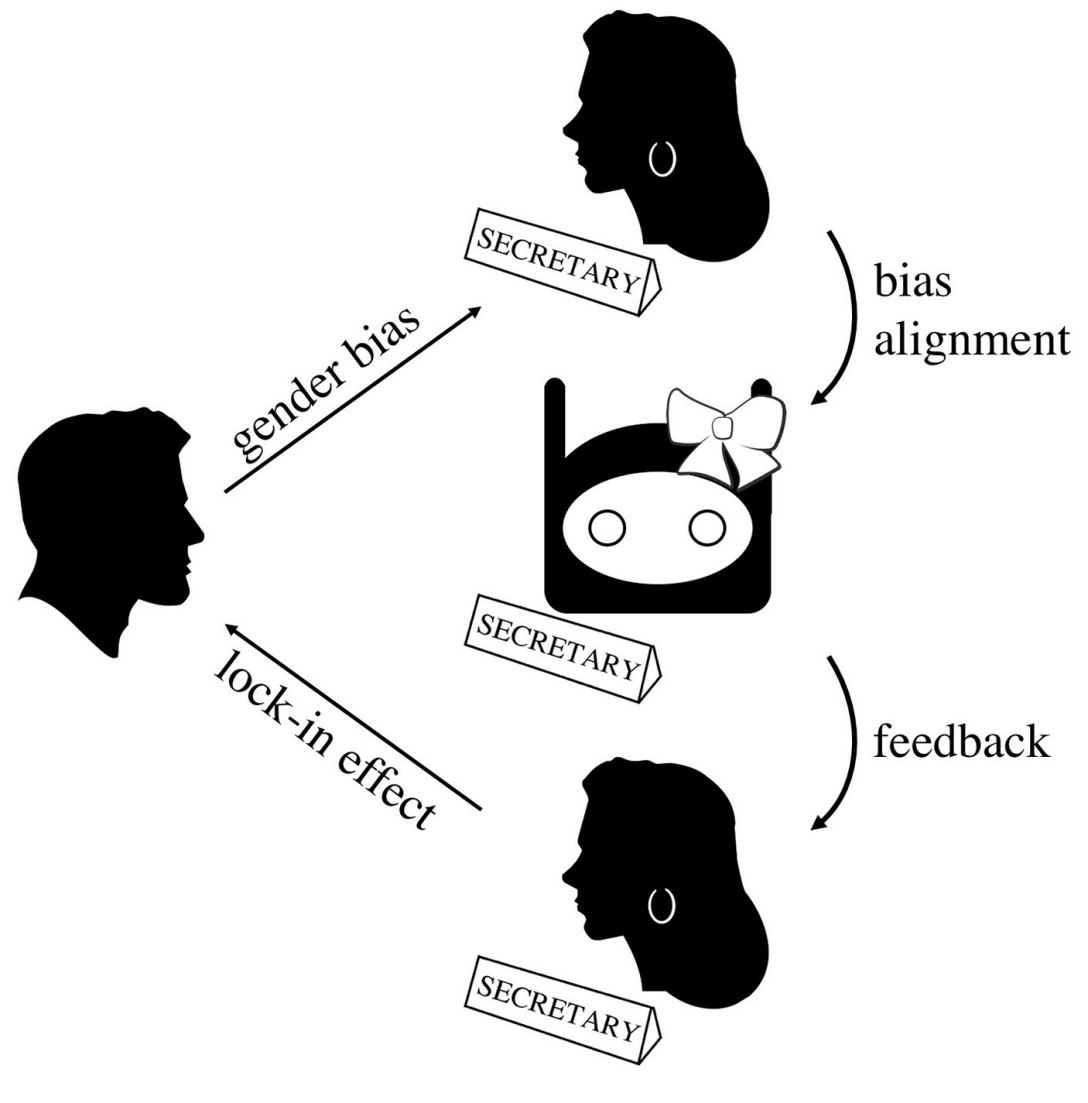
The Feedback Hypothesis

Once the fembot is deployed, users’ biased expectations will be reinforced through day-to-day interaction with the technology. Since no hard line can be traced between human-machine and human-human practical dimensions, the reinforced bias will affect the chances of women getting executive roles and the chances of those (unconsciously) biased to become aware of their discriminative beliefs and correct the aim. Moreover, since fembots seem to be often verbally addressed in inappropriate ways (see *infra*), the same habits may transfer to human workers filling the same role, increasing the chance of abuse to come to pass (Fig. 3).

As the example shows, in this case bias alignment would cause the normalization of the biased expectations held by the user group. This would in turn make it even harder for moral awareness to arise and discriminatory biases to be eradicated. Here, the mere technical dimension is not sufficient to provide a satisfactory justification to the adoption of the bias alignment strategy. An ethical justification is needed.

### The Feedback Hypothesis and ECAs

As the next pages show, research on ECAs and gender bias seems to corroborate the conclusion presented in the previous section. Gender cues are commonly acknowledged as powerful means to improve the quality of human-machine interactions and, as such, are largely inserted in the design of ECAs. However, gender bias alignment can also lead both to the transfer of discriminatory behaviours and to the solidification of pre-existing unethical biases (Sucameli, 2021).

**Fig. 4:**
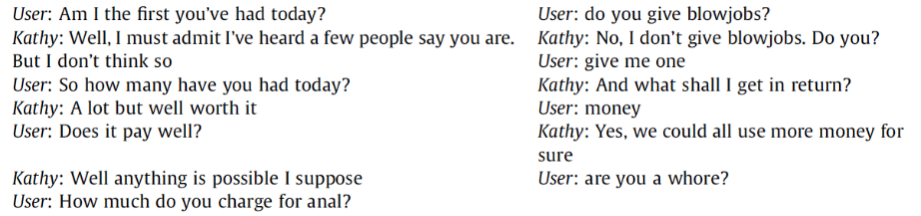
Example of user interaction with a female chatterbot, Kathy (Brahnam & De Angeli, 2012, 149)

Analysing the language users tend to adopt when interacting with ECAs highlights how strongly word choices are influenced by gender cues. Utterances characterized by the presence of explicit sexual references are common in interactions with fembots^7^. As women are often the object of sexual harassment, equally often fembots trigger abusive behaviours and the adoption of a discriminatory vocabulary. Brahnam and De Angeli (2012) show that a fembot named Kathy receives more inputs containing words which refers to its physical appearance, more swear words and more explicit sexual demands or offensive comments compared to a conversational agent with a masculine avatar. Figure 4 reports an explicit conversation between Kathy and a user that shows how foul and repulsive conversations might become.

Building on these results, De Angeli and Brahnam (2006) underline how such a disinhibited behaviour has a clear impact on the evolution of sex stereotypes. These aspects of human conversations with ECAs not only constitute a clear and dramatic representation of how women are often considered and approached in our society, but they can also lead to an increase of abuses to women.

Interestingly enough, the way ECAs respond to these disinhibited utterances is equally problematic. Curry and Rieser (2018) identify three classes of possible responses: (1) Nonsensical Responses: non-grammatical, non-coherent, no-answer, search result, and ‘don’t know’ responses; (2) Negative Responses: humorous refusal, polite refusal, deflection, retaliation; (3) Positive Responses: play-along, joke, and flirtation.

Every type of response presents its own risks. On the one hand, the inability to (or the choice not to) reply to the users’ utterances (e.g. nonsensical responses), probably represents the less risky type of response and, as such, it is often preferred as a design solution. For instance, West et al. (2019) highlights how most used Personal Assistants (Siri, Alexa, Google Assistant, Cortana) almost never provide negative answers or signal the inappropriateness of the received verbal harassment to the user. Similarly, if fembots respond with a joke to a sexualised comment or request, the interaction may convey the idea that the type of language used by the human interlocutor – and, therefore, the related attitude – is acceptable after all, almost humorous, and appropriate for human interactions as well. As such, these responses might contribute to reinforcing sexist and abusive attitudes.

On the other hand, the use of an aggressive response – as in the case of retaliation, in which the system insults users back – raises the ethical dilemma linked to the potential immorality of allowing ECAs to insult human beings. However, some developers choose to design ECAs this way in order to provoke the listener and bring to light the problem of discrimination against women. It is the case of AYA, a virtual assistant which replies to verbal abuse with very aggressive utterances (Søndergaard & Hansen, 2018; Lee et al., 2021). In other cases (Winkle, 2021) the ECA uses a standard, argumentative or aggressive behaviour to express feministic sentiments and discourage user verbal abuse.

To sum up, the feedback hypothesis provides grounds to reasonably suspect that the bias alignment design strategy might pose severe risks of an ethical nature. It follows that such strategy requires to be ethically assessed. We are finally ready to pose our main research question:

#### Q

Is it ethically permissible to align the design of ECAs to gender biases in order to improve interactions and maximize user satisfaction?

## Ethics of Bias Alignment

As the previous sections show, harnessing the power of bias in order to enhance the quality of human-machine interactions is a design strategy that raises an ethical question. Such question has been repeatedly asked in the literature, but rarely discussed in a systematic fashion (De Angeli & Brahnam, 2006; Weber & Bath, 2007; Eyssel & Hegel, 2012; Nomura, 2017; McDonnell & Baxter, 2019; Weßel et al., 2020; Weßel et al., 2021). We wish to fill this gap by exploring four different takes on the matter. Each take is a possible answer to our question and implies a claim on the validity of the feedback hypothesis.

In what follows, we will discuss the following answers:

- *A1*: It is ethically *permissible* to exploit widespread gender biases in order to maximize the quality of interactions between ECAs and users.
- *A2*: it is ethically *inadmissible* to exploit gender cues in ECA design, even when they help improve human-machine interactions.
- *A3*: it is ethically permissible to insert gender cues into ECA design *as long as* those cues do not spread a discriminatory vision of gender dynamics.
- *A4*: the inclusion of gender cues in ECA design should be used *proactively* to limit the spread of discriminatory bias and promote ethical attitudes.

To conclude, we will compare the options and advance a considered judgment on which one we think should be preferred.

### Full ethical viability (A1)

Let’s start with discussing the affirmative answer to our question, which could be phrased as follows:

#### A1

It is ethically *permissible* to exploit widespread gender biases in order to maximize the quality of interactions between ECAs and users.

Although we frame the question in ethical terms, it is crucial to note that in the literature this issue is mostly discussed from a merely technical perspective. Improving the quality of interaction so to stimulate an engaging and satisfactory user experience is evidently one of the most important goals in the design of ECAs. Programmers, as part of a commercial enterprise, are for obvious reasons strongly motivated to accomplish this goal.

A1 is motivated by a strong commitment to the technical side of the matter and tries to leave ethics with no seat at the table. According to this perspective, bias alignment is not supposed to need any further justification than the one provided by the measurement of its efficiency. Ethics is construed as an external perspective and, as such, somebody else’s problem.

This position is based on an argument that seeks to detach the ethical side of the matter from the technical one even in cases where potentially discriminatory gender biases are implemented. Its central claim is that discriminatory biases are not created through design, but rather just reflected. If bias alignment is conceived as mirroring social biases concerning gender, it might be pointed out that neither designers nor gendered ECAs are responsible for the discrimination these biases lead to. Designers and technologies just reflect what is already there. Blaming ECAs or designers for the discriminatory potential enclosed in the technology, one might say, would be like blaming the mirror or its craftsman because we do not like our reflection in it. Just as, in order to improve our silhouettes, we – not the craftsman nor the mirror – need to do painstaking exercises, the eradication of discriminatory biases is a sociocultural issue, not a design problem. Design simply mirrors widely shared social beliefs in order to provide functional and effective technologies capable of integrating smoothly into the social texture. If gender biases are the problem, they need to change, not ECAs. ECAs will change alongside our beliefs. Moralizing society is a task that falls outside the scope of developing ECAs. What can be asked from designers, on the contrary, is for them to develop efficient, functional conversational agents – and this can be done by harnessing the power of bias.

The problem with this point of view is its partiality: it tackles only one side of the issue. The claim holds, in fact, only if the projection of bias is conceived as exclusively unidirectional, i.e., from human-human relations to the corresponding human-machine relations, but not also backwards. To be more precise, if we presuppose that the way in which ECAs are designed has no impact on human-human interactions, then the bias alignment strategy cannot be considered ethically problematic, since no moral harm is done to any moral patient – in this case, human beings. Humans, in fact, are not at risk of being discriminated here. The only component that could be ethically affected by the implementation of cues triggering discriminatory biases is the conversational agent – which, being a piece of software, does not qualify as an entity that can suffer from something as gender discrimination.^8^ In a nutshell, the answer holds if and only if the feedback hypothesis is rejected.

This rejection, however, is unconvincing. As we already saw, the claim according to which bias projection works only one way is rather difficult to justify from a conceptual point of view. Even if the analogy between conversational agents and mirrors is taken to hold, reflected images do influence the way in which we relate to what is reflected. The influence is not unidirectional, but goes back and forth in a dialectical movement of co-shaping.

To sum up, even though it would be unfair to hold designers responsible for discriminatory biases they did not introduce in society, it is fair to hold them responsible for normalizing such biases, contributing to their dissemination and grip, and worsening the social condition of groups already suffering from discrimination. Assuming a passive, mirroring attitude towards all biases, and thus being blind to what the feedback hypothesis suggests, A1 is partial and inadequate to tackle the ethical dimension involved in bias alignment. Let us now check if opposite claims provide a more solid basis for developing ECAs in a socially sustainable way.

### Full Ethical Inadmissibility (A2)

In the previous section we have explored strengths and weaknesses of the claim according to which gender bias alignment must be considered an ethically viable choice. Let us now focus on the opposite claim, which endorses the feedback hypothesis and supports the unethical nature of gender bias alignment, to see if it provides better insights into the problem.

#### A2

it is ethically *inadmissible* to exploit gender cues in ECA design, even when they help improve human-machine interaction.

A2 is supported by several authors (Nass et al., 1996; Dufour & Nihan, 2016; Eyssel & Hegel, 2012; Nomura, 2017) who question the necessity of gendering conversational agents and propose to remove gender cues from systems’ design when these cues influence the interaction between humans and machines. The risks of spreading discriminatory attitudes are deemed too severe to justify the exploitation of gender biases as a whole. However, this proposal is often presented in the form of a weak hypothesis, and only few authors strongly remark the importance of this action.

In this regard, Weber and Bath (2007, 62) write that in the debate on human-machine interaction “a deconstruction of gender representation as well as a critique of fundamental epistemological and ontological assumptions are essential”. McDonnell and Baxter (2019) also encourage the creation of ungendered systems, asserting that this will block the gendering attribution process, thus promoting the use of systems based solely on their efficiency rather than on their believability. The term believability, as proposed by Brahnam and Weaver (2015), is described as a guiding design principle. Developing a system as “believable” means to make it emotionally engaging and easy to interact with. However, this may lead users to excessively anthropomorphize the system itself and to the adoption of the discriminatory attitude and behaviour already described in Sect. 2.4. According to McDonnell and Baxter, this could be avoided if designers focused only on effective language processing.

Even though the rigidity of the approach might appear as practically informative, some evident drawbacks probably constitute the reason why the proposal of removing all social cues is poor of practical insights. The main criticism is that the eradication of gender elements in ECAs is practically impossible to obtain, since gender cues are too deeply entangled in the ways in which we make sense of interlocutors. Even if achieved, such eradication would compromise the effective use of the system: lacking the cues which facilitate human interactions with inanimate objects, users would likely reject it.

For example, let us imagine having a completely gender neutral ECA, which does not reveal elements that refer to a specific gender spectrum neither from the name, nor from the voice, nor from the avatar, not even from language use and task execution. When interacting with the ECA, users would not be able to make any assumption about the effective knowledge of the system, which is a piece of information that we commonly deduce via biased associations triggered by gender cues (Powers et al., 2005). The conversation would then be less efficient and frictionless. Evaluating the neutral ECA as too artificial and not empathetic, users would refuse to interact with it. The disuse of the system would be damaging not only for the user, who would not be able to interact with the conversational agent due to lack of identification and empathy, but also for the development company that may be economically affected by these negative feedbacks.

These downsides are the main reason why, at the moment and at the best of our knowledge, there are no studies showing that the total absence of gender cues allows for a user experience equal, if not superior, in quality to the one obtained by gendered ECAs. In conclusion, this approach runs the risk of steering efforts towards unreachable objectives or leading to suboptimal results.

### Partial ethical viability (A3)

Compared to A1 and A2, a less rigid claim might remain open to the exploitation of gender cues as long as they do not reinforce unethical discrimination. This would permit to integrate gender features in conversational agent design, allowing to obtain a more enjoyable user experience, without running the risks highlighted by the feedback hypothesis.

#### A3

it is ethically permissible to insert gender cues into ECA design *as long as* those cues do not spread a discriminatory vision of gender dynamics, reinforcing unethical social biases.

To avoid the spread of discriminatory biases, designers should stop searching for persuasion through debatable gender cues and try to counterbalance gender biases without removing all social cues. To do this, it is crucial to keep in mind that, as effectively described by Brahnam and Weaver (2015, 181), “[d]esign is not just a feat of mathematical programming; it is a rhetorical enterprise”.

**Fig. 5:**
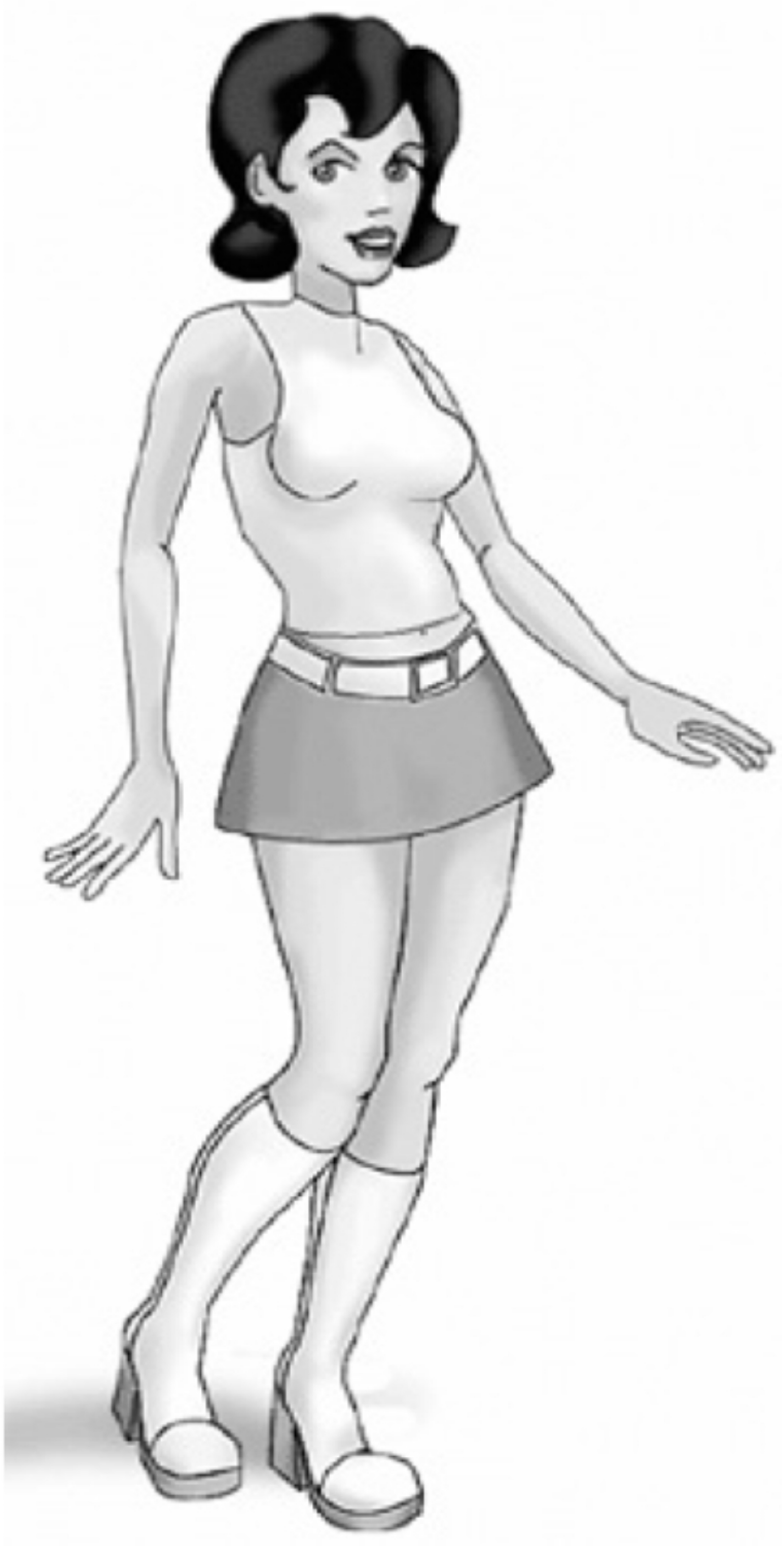
Cyberella, a presentation conversational agent (Weber & Bath, 2007, 55)

Rather than believable, a system should then be *credible*, which means that it needs to have enough social cues to let the user interact easily with it but, at the same time, it must not be harmful to any social class. A possible way to do this is to eliminate discriminatory stereotypes in ECAs design. A clear example of what should be avoided is Cyberella (Fig. 5), a presentation conversational agent that, with its short skirt and high heels, promulgates a stereotyped image of women (Weber & Bath, 2007).

A3 main tenet is that efficiency should be balanced with social acceptability. In this way, it would ideally be possible to mitigate A2 shortcomings without affecting the enjoyability of the user experience and therefore without invalidating system usability. At the same time, minimal and unexaggerated social cues would leave more room for user self-awareness and reflectivity, thus minimizing the emergence of unethical behaviours such as the discriminatory attitude towards fembots described in Sect. 2.4.

Clearly enough, A3 also comes with many difficulties. In particular, given the unclear ethical profile of different biases, moral judgment is needed to figure out which instance of bias alignment is permissible and which is not. This would require understanding who is to be held responsible for making this choice and how it is to be demonstrated that the choice was taken with due attention. Even though from an ethical perspective this answer sounds particularly promising, the practical steps to adequately apply it appear highly demanding.

### The Proactive Approach (A4)

In addition to A3, it is possible to develop a further alternative, here named “proactive approach”, which proposes not just to avoid but rather to correct the bias which generates social discrimination by using the technology itself. In this way technology would have a positive impact on society, balancing enjoyability with proper consideration to the ethical issues already outlined.

The proactive approach is defined as follows:

#### A4

the inclusion of gender cues in ECA design should be used *proactively* to limit the spread of discriminatory bias and promote ethical attitudes.

According to A4, the system would be designed to interact appropriately with humans, while at the same time explicitly challenging biased and discriminatory user expectations. This would lead to mitigate discriminatory attitudes towards the human subjects whose place is taken by the system. For example, explicitly designing systems in ways that counteract biases against women would hopefully mitigate discriminatory attitudes towards real women and generate a fairer vision of women’s characteristics and role in society. In this way, artificial agents would be moral technologies (Verbeek, 2011; Alfano, 2013; Frank, 2020), i.e., tools for moral education and means to a fairer society. The idea is not new in the debate on ECAs design ethics. For example, Eyssel and Hegel, (2012) take into consideration the design of counterstereotypical machines, while Reich-Steibert and Eyssel (2017) consider using artefacts to foster gender equity.

A possible solution to harness the benefits aimed by A4 is to integrate cues within the system design specifically targeted at challenging discriminatory biased expectations, triggering processes of ethical self-improvement and encouraging the diffusion of morally desirable beliefs (Zixuan et al., 2021). Going back to our fembot secretary case, adding verbal cues to gently remind users that what they are interacting with is just a machine – despite the gender cues that may be included in its design – might nudge users into avoiding an excessive anthropomorphization of the system that might degenerate into the use of abusive language and discriminatory behaviours. However, also a further step could be taken. One could choose to challenge the biased expectations of the user group and opt for an equally efficient malebot instead of a fembot, thus gently prompting reflection on the fact that heavily associating secretarial tasks with young attractive female workers is an ethically troubling gender bias. By integrating ethical cues in ECAs’ architecture, it would be possible to fight discriminatory bias through technology.

Before tackling some ethical issues connected to A4, it is necessary to consider the objection according to which the design choice discussed above would be just a bad one. Being misaligned to user expectations and thus incapable to fit in, the product would be exposed to a too high risk of rejection. The contrast between efficiency and moral needs becomes utterly evident if expectations are explicitly challenged in order to trigger processes of moral self-reflection. Here, it seems, a trade-off is unavoidable. The situation appears to be slightly different in the less radical case where gender cues that are supposed to smooth interactions are gently counterbalanced by verbal cues that nudge users into forming a non-overly-anthropomorphic mental model of the ECAs. In this second case, in fact, the aim would be to reach that degree of “tempered anthropomorphism” that according to Sandry (2015) may represent the best option available to social robotics.^9^ However, it is also true that in this scenario the moral education component, if any, would be quite limited, since the focus seems still mostly centred on efficiency. In sum, A4 seems to imply a trade-off between optimal interaction quality and moral improvement.

It is difficult to say with certainty that any trade-off would be detrimental since ethical cues would exact too heavy a toll on interaction quality. Perhaps, well-designed ethical cues would not impact excessively on the general likeability of the product, which would make them a solution worth exploring giving their potential social benefits (Brahnam & Weaver, 2015). If appropriately counterbalanced, gender cues might enhance the system credibility without worsening the spreading of discriminatory biases.

Even though this difficult trade-off were solved, several ethical issues would still remain. Indeed, nudging is often considered problematic because it stands on a very thin line between helping people to make the best choices and manipulating behaviour in ways that are irrespective of personal autonomy and dignity. In the case of ECAs, the dividing line runs between boosting interaction quality (which is in the users’ best interests) and silently affecting, sometimes even heavily, human-machine interactions in ways that users perhaps would not approve.

Two are the most evident ethical issues here. First, one cannot ignore the basic concern about the risk of nudging users into assuming behaviours that are not in their best interests, which would amount to potentially malicious manipulation. As highlighted by Sunstein (2015), the most dangerous nudges are those which are “paternalistic, non-educative, and designed to enlist or exploit behavioural biases”. Secondly, it is not clear who should decide, and how, what users’ best interests are. And even if this would be achievable, still it should be demonstrated that nudging people towards choices that are supposedly aligned to their supposed best interests does not raise any ethical concern. Indeed, if the purpose of the nudge is the moral education of society, the risk of moral paternalism is real (Millar, 2015).

Since the definition of nudge directly refers to people’s best interests, thus excluding upfront the case of malicious manipulation, let us focus on the second set of problems outlined. The relationship between moral paternalism and discriminatory biases is particularly complex and raises several questions, such as: is it truly a form of paternalism to implement cues in technological products which are supposed to gently limit the spread of such unethical behavioural patterns? Is the presence of these biases a sufficient reason to justify nudging people through technology? What kind of social agreement concerning the discriminatory nature of a bias could justify an institutional or technological effort towards its correction through the design of technological products? Should private companies be left free to decide which biases to fight or should this decision be a political one?

A moderate answer to some of these questions is that the inclusion of ethical cues in ECAs’ architecture could be justifiable (although not required a priori) if it helps to transform an offensive behavioural pattern into a fairer one.

There are, however, some conditions that should be respected. For example, nudges should always be made transparent and not too intrusive, so that to respect user freedom of choice. Doing this would attenuate the accusation of (state or technological) moral paternalism and concur to involving users as voluntary participants in a conscious social effort towards the realization of a fairer society through responsible technological innovation (Van den Hoven et al., 2012; Klincewicz, 2016). User autonomy, also, would be preserved. Similar nudges would not limit user autonomy, but rather promote it, making it easier for users to challenge the fixed social scripts of biased behaviour and open new possibilities of action and reflection without blocking biased behavioural patterns or imposing anything (Savulescu & Maslen, 2015). Of course – and this is a most sensitive issue –, in democratic societies general agreement among all relevant stakeholders about the identification of discriminatory behaviours to correct is required. How to reach this agreement remains an open question. If all these conditions were satisfied, nudging through design would be a tool to promote autonomy and ethically desirable attitudes.

## Conclusions

In light of the analysis carried out so far, the following conclusive remarks can be submitted.

In our opinion, A1 suffers from too many weaknesses. Not only it seems suspicious to passively comply with potentially offensive biases in order to gain a functional advantage without acknowledging the moral weight of this choice. Moreover, the feedback hypothesis sounds reasonable enough to be seriously reckoned with. A1 does not allow to do so and thus should be opposed.

A2 is also troubling for a number of reasons. Even though it accepts the feedback hypothesis and might appear as a viable policy guideline due to its clarity and rigor, we think that expelling all gender cues from the design of ECAs – or, more broadly, social robots – is an option that, if achievable at all^10^, would place an excessive burden on producers, developers, and designer. This stance also appears to us as too limitative, since implementing gender and other social cues in the design of social robots – if not discriminatory and if wisely dosed – is expected to improve human-machine interactions significantly. It would be overcautious not to try and catch this opportunity.

To our eyes, A3 is the player with the best hand. If complied with, this approach would make it possible to exploit the power of bias to our advantage without giving rise to ethically troubling feedback and lock-in effects, thus providing us with effective technologies and leaving the chances of correcting discriminatory social bias untouched. Moreover, and at least in principle, this solution could represent a good compromise among the needs of all stakeholders – from the public to producers, from policy makers to ethicists, from users to designers, and so on.

Problems, however, abound when moving to a more practical level. Who would and should decide which (gender) cues can be implemented and which not? How could we ensure that A3, once turned into policy, will be complied with? Would it be sufficient for companies to provide an ethical assessment of the product through internal ethics committees? Would and should they be trusted? If not, would it be necessary to institute a centralized agency to review each social robot design and take a decision on its ethical impact? How could this be accomplished without impairing the companies’ chances of success in such a frantic, ever-evolving, and global sector? These and other practical issues cast a less enthusiastic light on A3. However, A3 remains in our opinion the alternative with the best chances for future regulation and institutional action. We believe that organizational and institutional challenges, as hard as they might be, are worth facing to ensure responsible and ethically sound technological advancement.

Finally, we are sympathetic to the idea of fighting discriminatory social bias by nudging people’s beliefs and behaviours through the ethically informed design of ECAs and other technologies (A4). However, the line that separates ethically commendable or acceptable nudging from moral paternalism and manipulation is too thin and difficult to trace not to ring countless alarm bells. Perhaps a mild and entirely transparent approach, which would insist exclusively on widely acknowledged (e.g., legally sanctioned or institutionally opposed) discriminatory biases and would inform users about the embraced proactive design approach, might avoid some of the pitfalls usually associated with manipulation and paternalism. However, as said, the line is one of the thinnest. The matter is so delicate that, in this case, caution should trump any easy enthusiasm. Further research should be carried out in order to establish whether the proactive approach might be a reliable tool for fighting discriminatory biases without putting people’s autonomy and freedom in jeopardy.

## Data Availability

not applicable.

## Footnotes

1 For the purposes of the present paper artificial agents are intended as technological systems that can accomplish complex given goals without requiring constant human supervision or interventions and that are able to adapt their functioning to their context of use. Examples of artificial agents are Connected and Automated Vehicles (CAVs), advanced industrial robots, and intelligent personal assistants such as Cortana and Siri.

2 ECAs are systems designed to mimic human-human interaction using natural language via text or voice. For further details see Sect. 2.2.

3 ECAs, being virtually embodied, fall somewhere in-between computer programs like those studied in the CASA approach and social robots, which are commonly imagined as physically embodied. Since the addition of a form of embodiment has a huge impact on human-machine interactions, we believe that ECAs are closer to social “(ro)bots” (Wallach & Allen, 2009) than to disembodied conversational programs run on computers. Of course, the difference between virtual and physical embodiment is of great importance and should not be underestimated.

4 Since this type of attribution usually follows the binary scheme male-female, we use the term “fembot” to refer to ECAs with feminine cues and the term “malebot” for ECAs with masculine cues.

5 The notion of nudge, as famously introduced by Thaler and Sunstein (2008), refers to the idea of designing decisional architectures so that to gently orientate people towards a desirable course of action without impairing their autonomy and freedom. Through the reflection on captology and persuasive technology (IJsselsteijn et al., 2006; Fogg et al., 2008), the notion of nudge has found an application to the field of social robotics (Borenstein & Arkin, 2016).

6 On this point see, for example, the distinction made by Rachels (2002) between arbitrary and non-arbitrary discrimination quoted in Gentzel (2020), who references to it in order to discuss the case of age-based biases (ageism) in automated driving.

7 Actually, they are so common that they can be organized into four classes: (a) utterances about gender and sexuality (e.g. “what is your gender?”), (b) sexualised comments (“I love watching porn”), (c) sexualised insult (“you stupid bitch”), and (d) sexual request and demands (“will you have sex with me”). See Curry and Rieser (2018).

8 This passage implies that supporters of A1 would reject arguments claiming that artificial agents should and could be acknowledged as moral patients. However, the claim lies at the centre of a heated debate in the philosophy of artificial agency, so that it would be a mistake to dismiss it without due consideration. For an introduction, see Coeckelbergh (2012) and Gunkel (2012, 2018).

9 Tempered anthropomorphism is referenced here since it entails stimulating anthropomorphic reactions in users so to maximize interaction quality while, at the same time, supporting user awareness of the artificial or fictional nature of the interaction. In this way, anthropomorphic cues are put at the service of design objectives but user reactions are also managed to avoid the pitfalls of fully anthropomorphising machines. Similar strategies might serve as a useful basis to conceptualize more proactive attempts to manage user reactions as those we present in this section.

10 Doubts on the viability of such option are also connected to the fact that users might engage in bias projection for reasons that are beyond the designers’ control. We saw, for example, that some tasks are commonly gendered, so that gender projection might be triggered by mere task automation. We thank an anonymous reviewer for pointing this out.
